# Effect of Different Proteases on the Degree of Hydrolysis and Angiotensin I-Converting Enzyme-Inhibitory Activity in Goat and Cow Milk

**DOI:** 10.3390/biom8040101

**Published:** 2018-09-27

**Authors:** Guowei Shu, Jie Huang, Chunju Bao, Jiangpeng Meng, He Chen, Jili Cao

**Affiliations:** 1 School of Food and Biological Engineering, Shaanxi University of Science and Technology, Xi’an 710021, China; huangjie9319@gmail.com (J.H.); lhm542527328@163.com (C.B.); chenhe419@gmail.com (H.C.); 2 Department of Research and Development, Xi’an Baiyue Gaot Milk Corp., Ltd., Xi’an 710089, China; byjpmeng@gmail.com; 3 Department of Research and Development, Xi’an Oriental Dairy Co., Ltd., Xi’an 710027, China; xiandfcjl@gmail.com

**Keywords:** angiotensin I-converting enzyme (ACE)-inhibitory peptides, alkaline protease, degree of hydrolysis, enzymatic hydrolysis, goat and cow milk

## Abstract

Angiotensin I-converting enzyme (ACE) peptides are bioactive peptides that have important value in terms of research and application in the prevention and treatment of hypertension. While widespread literature is concentrated on casein or whey protein for production of ACE-inhibitory peptides, relatively little information is available on selecting the proper proteases to hydrolyze the protein. In this study, skimmed cow and goat milk were hydrolyzed by four commercial proteases, including alkaline protease, trypsin, bromelain, and papain. Angiotensin I-converting enzyme-inhibitory peptides and degree of hydrolysis (DH) of hydrolysates were measured. Moreover, we compared the difference in ACE-inhibitory activity between cow and goat milk. The results indicated that the DH increased with the increase in hydrolysis time. The alkaline protease-treated hydrolysates exhibited the highest DH value and ACE-inhibitory activity. Additionally, the ACE-inhibitory activity of hydrolysates from goat milk was higher than that of cow milk-derived hydrolysates. Therefore, goat milk is a good source to obtain bioactive peptides with ACE-inhibitory activity, as compared with cow milk. A proper enzyme to produce ACE-inhibitory peptides is important for the development of functional milk products and will provide the theoretical basis for industrial production.

## 1. Introduction

In recent years, bioactive peptides have become a hot research topic in the field of food and medicine [1,2]. Most of these peptides have a small molecular mass, allowing for easy digestion and absorption of protein by the human body. These bioactive peptides can provide nutrition for human growth and development, regulate the physiological functions of the human body, and play a role in prevention and treatment of diseases [1,2,3]. Angiotensin I-converting enzyme (ACE)-inhibitory peptides are bioactive and have been widely studied in the past few decades. The ACE is a key enzyme associated with the renin–angiotensin system, which regulates peripheral blood pressure. It catalyzes both the production of vasoconstrictor angiotensin-II from angiotensin-I and the inactivation of the vasodilator bradykinin, which consequently results in hypertension [4]. Inhibition of angiotensin-II’s formation by ACE-inhibitors has proved to be successful in the treatment of hypertension and related target organ damage [5]. However, the use of ACE-inhibitory drugs is reported to cause adverse side effects such as hypotension, cough, increased blood calcium levels, fetal abnormalities, reduced renal function, angioedema, and skin rashes [4,6]. Compared to chemosynthetic drugs, ACE inhibitory peptides derived from natural sources such as food proteins are believed to be safer for consumption and have fewer adverse effects [7]. People have successfully isolated a variety of ACE-inhibitory peptides from food proteins [8], plant proteins [9], marine biological proteins [8], and other resources, and have confirmed the inhibitory effect on ACE activity through animal experiments and clinical trials.

The casein and whey proteins found in milk are a good source of the bioactive peptides that have a positive impact on body functions. These peptides are inactive within the sequence of precursor proteins but can be released in vivo or in vitro by enzymatic hydrolysis [10] or during fermentation with lactic acid bacteria [11]. Other methods have also been confirmed for releasing bioactive peptides, such as chemical synthesis [12], extraction from natural foods [13], and DNA recombinant technology [14]. Enzymatic hydrolysis is one of the most commonly used methods for the preparation of ACE-inhibitory peptides from food proteins [15], as it has several advantages. It is of low cost in simple conditions, causes little damage to the nutritional value of proteins, and represents an easy-to-control hydrolysis process. Most importantly, through this process a specific type of peptides is produced by hydrolyzing proteins at a specific position. Since the structure and activity of bioactive peptides can be affected by the method of production, there is a need to evaluate the efficiency of proteases in releasing ACE-inhibitory peptides from milk proteins.

Cow milk is a highly nutritious type of food; it has a higher concentration of folate [16] and lower fat content than goat milk [17]. However, some studies suggest that goat milk has higher nutritional value than cow milk. Goat milk has higher concentrations of phosphorous, potassium, vitamin A, and calcium [18]. Additionally, it is suitable for lactose-intolerant patients [19]. To better understand the efficiency of ACE-inhibitory peptides from goat and cow milk, we evaluated the ability of several proteases to produce ACE-inhibitory peptides from the two types of milk. In addition, we further compared the difference of ACE-inhibitory activity between the two types of milk. A proper enzyme to produce ACE-inhibitory peptides is important for further development of active functional foods and will provide a theoretical basis for industrial production.

## 2. Materials and Methods

### 2.1. Materials and Chemicals

Skimmed cow and goat milk powder were obtained from Anchor, Fonterra Co-operative Group (Waikato, New Zealand). Hip-His-Leu (HHL) and ACE were purchased from Rosen Technology Co., Ltd. (Xi’an, China). Alkaline protease, trypsin, bromelain, and papain were purchased from Sigma-Aldrich (St. Louis, MO, USA). Other inorganic regents were purchased from the Hongyan Chemical Reagent Factory (Tianjin, China).

### 2.2. Preparation of Enzymatic Hydrolysates from Cow and Goat Milk

Skimmed cow and goat milk powder were mixed with distilled water at a ratio of 5:100 (*w*/*v*), respectively. Then, four different enzymes (alkaline protease, trypsin, bromelain, and papain) under their optimal conditions (shown in Table 1) were inoculated into reconstituted skim milk pasteurized with 5% inoculum, respectively [20]. During enzymatic reactions, 0.1 mol NaOH was added to maintain pH at the optimal level, and the solution was continuously stirred for 6 h. The hydrolysis reactions were terminated by heating at 90 °C for 15 min in water bath. Finally, the temperature and pH of hydrolysates were adjusted to 25 °C, and 7.0, respectively. The hydrolysates catalyzed by four proteases were centrifuged at 10,664× *g* for 15 min (Model GL21, Instrumentation, Hunan, China), and the supernatant was collected to evaluate ACE-inhibitory activities [21].

### 2.3. Assay of Degree of Hydrolysis

Four proteases were added to substrate in water bath under their optimal temperature and pH. During enzymatic reactions, a solution of 0.1 M NaOH was added dropwise at regular intervals to keep the pH constant. The volume of the NaOH consumed was recorded and then the degree of hydrolysis (DH) of the protein was determined according to the pH-Stat method [22]. The formula is as follows:

(1) $$\mathrm{Degree}\;\mathrm{of}\;\mathrm{hydrolysis}\;(\mathrm{DH},\;\%)=\frac{B\times Mb}{\alpha \times Mp\times h_{tot}}\times 100\%,$$

where B is the volume of NaOH (mL) and Mb represents 0.1 mol NaOH. Alpha (α) is the reciprocal of the DH of casein hydrolysis reaction (α = 0.442), Mp is the quality of protein, and h_tot_ is the amount of peptide bonds per mass unit of protein (h_tot_ values for goat and cow milk were 8.35 mmol/g and 8.29 mmol/g, respectively).

### 2.4. Assay of Angiotensin I-Converting Enzyme-Inhibitory Activity

The ACE-inhibitory activity of hydrolysates was measured based on the method of Cushman et al. [23] with some modifications. Here, 200 μL of HHL buffer (5 M HHL dissolved in 0.1 M sodium borate buffer containing 0.3 mol/L NaCl, pH 8.3) were mixed with 100 μL of enzyme solution and pre-incubated for 5 min at 37 °C. The reaction was initiated by adding 20 μL of ACE (dissolved in borate buffer, 0.1 UN/mL), and the mixture was incubated for 30 min at 37 °C. Finally, the mixture was terminated by adding 250 μL of 1.0 N HCl, using the same volume of distilled water instead of sample and ACE in the control and the blank group, respectively. Then, the three mixtures were respectively mixed with 1.7 mL of ethyl acetate. The liberated hippuric acid was extracted with ethyl acetate, and 1.0 mL of ethyl acetate was removed from the ethyl acetate layer. Three materials were dried at 120 °C for 30 min and redissolved in 1.0 mL of distilled water. The absorbances of the sample group, the control group and the blank group were measured at 228 nm using a spectrophotometer (Shanghai Spectrum Instruments Co., Ltd., Shanghai, China). The average value of three measurements was used to calculate the ACE-inhibitory rate. The extent of inhibition was calculated as follows:

(2) $$\mathrm{ACE}-\mathrm{inhibitory}\;\mathrm{activity}(\%)=\frac{O_{b}-O_{a}}{O_{b}-O_{c}}\times 100\%,$$

where O_a_ represents absorbance of ACE and ACE inhibitors existing at the same time (sample group); O_b_ represents absorbance with ACE and without ACE inhibitors (control group); and O_c_ represents absorbance without ACE and without ACE inhibitors (blank group).

### 2.5. Statistical Analysis

All experiments and measurements were performed three more times. Statistical analysis was performed using the Origin 9 software package (Origin Lab Inc., Alexandria, VA, USA) and Microsoft Excel 2010 (Redmond, WA, USA).

## 3. Results and Discussion

### 3.1. Effect of Four Different Proteases on the Degree of Hydrolysis of Hydrolysates from Goat and Cow Milk

**Figure 1:**
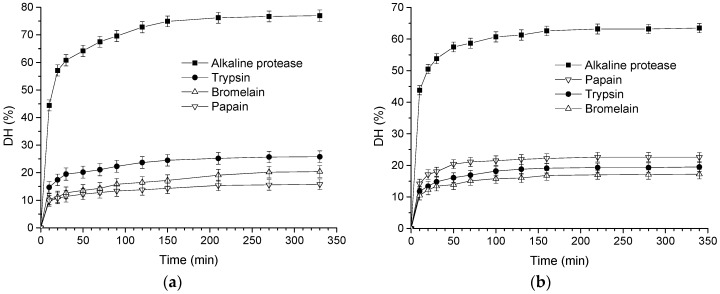
Effect of four proteases on the degree of hydrolysis (DH) from skimmed milk: (**a**) goat milk and (**b**) cow milk. Hydrolysis conditions were as follows. Alkaline protease 5%, 45 °C, pH 9.5; trypsin 5%, 37 °C, pH 8.0; bromelain 5%, 45 °C, pH 6.0; papain 5%, 37 °C, pH 7.0. The error bars represent standard deviation of means (*n* = 3).

Skimmed goat and cow milk were hydrolyzed by four proteases including alkaline protease, trypsin, bromelain and papain, which correspondingly produced different hydrolysates. Figure 1 shows the changes in DH of the hydrolysates during the course of hydrolysis. All hydrolysis curves increased rapidly in the first 50 min; then the hydrolysis speed of four enzymes remained almost unchanged. Figure 1a suggests that the highest DH of hydrolysates from goat milk was produced with alkaline protease (77.0%) at a 330 min reaction time, followed by trypsin (25.8%) and bromelain (20.4%); the DH of hydrolysate using papain was only 15.8%. The DH for cow milk hydrolysates catalyzed by alkaline protease, trypsin, bromelain, and papain reached maximum values of 63.5%, 19.5%, 22.6%, and 17.2%, respectively, at 340 min.

The DH of hydrolysates increased quickly at the beginning and then kept stable with the increase of hydrolysis time. The rate of hydrolysis decreased, perhaps owing to reduced number of peptide bonds, resulting in the proteases and their substrates reaching a state of saturation [21]. Among different enzyme treatments, the alkaline protease-treated hydrolysates showed significantly higher DH values than did the other different enzyme treatments, indicating that the alkaline protease has a more extensive ability to hydrolyze the milk proteins than trypsin, bromelain, and papain. The values of DH differed by enzyme treatment due to the difference in the catalytic sites of endopeptidases and exopeptidases [24]. Alkaline protease is a member of the serine S8 endoproteinase family, which has a broad specificity, and therefore exhibited more binding sites compared with the other enzyme treatments. Alkaline protease-assisted reactions have been reported to exhibit higher DH compared to neutral or acid enzymes from plant, animal, or microbial origin, such as papain, pepsin, and neutrase, respectively [25], consistent with the results of this study. The lower DH of the bromelain and papain hydrolysate could also be due the fact that the pH values used for hydrolysis with these enzymes (6.0 and 7.0) approach the isoelectric point of the primary whey proteins (5.2) and the caseins (4.6), and could have resulted in some aggregation and masking of susceptible peptide bonds [26].

### 3.2. Influence of Four Proteases on the Angiotensin I-Converting Enzyme-Inhibitory Activity of Hydrolysates from Goat and Cow Milk

**Figure 2:**
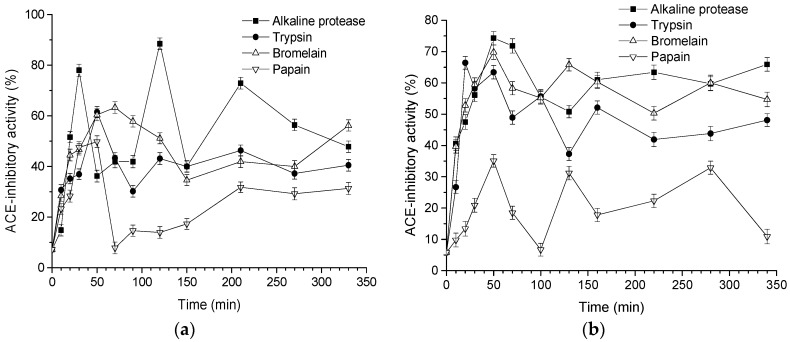
Influence of four proteases on the angiotensin I-converting enzyme (ACE)-inhibitory activity of hydrolysates from skimmed milk: (**a**) goat milk and (**b**) cow milk. The error bars represent standard deviation of means (*n* = 3).

Different proteases have their own specificity for proteins, and the hydrolysis positions are also different. Four proteases were used to hydrolyze goat and cow milk, and their corresponding hydrolysates’ ACE-inhibitory activity was measured. Figure 2 indicates that the ACE inhibitory activity values of unfermented goat and cow milk at the initial stage were 7.2% and 5.8%, respectively. With the increase of hydrolysis time, hydrolysis with alkaline protease, trypsin, bromelain, and papain significantly increased the ACE-inhibitory activity. However, the curves of ACE-inhibitory activity catalyzed by four enzymes were unstable for both skimmed goat and cow milk, even though the DH of hydrolysates rose with the increase in hydrolysis time. Higher DH may result in further degradation of ACE-inhibitory peptides into short peptides with more or less activity [27]. As the hydrolysis progresses, the enzyme continues to cleave the peptides, and subsequently certain amino acids at the C- or N-terminus that usually have strong affinity with the active site of ACE may be further cleaved, resulting in smaller and weaker peptides. Occasionally, enzymatic hydrolysis will produce peptides with increased ACE-inhibitory activity if the new peptides have C- or N-terminal amino acid residues with a strong affinity towards ACE [28]. The maximum ACE-inhibitory activity of hydrolysates produced by alkaline protease, trypsin, bromelain, and papain for goat milk were obtained at 120 min (88.5%), 50 min (61.5%), 70 min (63.3%), and 50 min (49.8%), respectively (Figure 2a). As for cow milk, the greatest ACE-inhibitory activity for alkaline protease, trypsin, bromelain, and papain was observed at 50 min, with values of 74.3%, 63.4%, 69.8%, 35.1%, respectively (Figure 2b). It can be concluded that alkaline protease is the best protease to produce ACE-inhibitory peptide for both goat and cow milk. A study investigated the effect of alcalase, pepsin, and trypsin on hydrolysates from chicken bones and found that the hydrolysates catalyzed by alcalase had the highest peptide content and DH [29]. Another study also observed that the alkaline protease-treated hydrolysates of soybean meal had a higher ACE-inhibitory activity than that of neutral protease, papain and trypsin [30]. Similarly, ovomucin hydrolyzed with alcalase produced bioactive peptides with high ACE-inhibitory activity [31]. In the present study, we found that ACE-inhibitory activity of goat and cow milk catalyzed by papain was lower than that of other enzymes. Huang et al. [32] used papain to hydrolyze ovalbumin and found that the ACE inhibition rate of hydrolysates reached 70.6 ± 1.1% which was significantly higher than in this study (goat milk, 49.8%; cow milk, 35.1%), indicating that there are fewer binding sites between papain and the two types of milk. In addition, the ACE-inhibitory activity of bromelain was higher than that of papain. This might be due to bromelain catalyzing essential groups that were not sugar molecules but the thiol of the peptide chain. Additionally, the goat and cow milk catalyzed by the alkaline protease obtained the maximum values of ACE-inhibitory activity, at 88.5% and 74.3%, respectively, and the alkaline protease-treated hydrolysate showed the highest DH (Figure 1). Therefore, goat milk is a good source for obtaining bioactive peptides with ACE-inhibitory activity, as compared with cow milk.

### 3.3. Relationship between the Degree of Hydrolysis and Angiotensin I-Converting Enzyme-Inhibitory Activity

Figure 1 and Figure 2 clearly show that the unhydrolyzed protein substrates had very low ACE-inhibitory activity (<8% inhibition). In order to release ACE-inhibitory peptides from the sequence of milk proteins, hydrolysis was necessary. The DH values observed for the different hydrolysates continuously increased, while the trends of ACE-inhibitory activity were almost unaffected by further increases in DH. In many cases, the values for DH and ACE inhibition activity was enhanced with the increase of hydrolysis time. One study has reported that the correlation between ACE-inhibitory activity and DH could best be described by a logarithmic function with the equation *y* = *a* − (*bc^x^*), where *y* is the ACE-inhibitory activity, *x* is the DH, and *a*, *b*, and *c* are the regression parameters [33]. The goat milk-derived ACE-inhibitory activity by alkaline protease changed from 14.8 to 88.5%, by trypsin it changed from 30.2 to 61.5%, by bromelain it changed from 28.4 to 63.3%, and by papain it changed from 7.8 to 49.8%. However, the DH changed only slightly with the four enzymes, by 44.4–77.0%, 14.7–25.8%, 10.4–20.4%, and 9.8–15.8%, respectively. In addition, the DH of the cow milk-derived hydrolysates by alkaline protease, trypsin, bromelain, and papain changed from 43.8%, 14.2%, 11.8%, and 10.5 to 63.5%, 19.5%, 22.6%, and 17.2%, respectively, while their corresponding ACE-inhibitory activity values were 40.6–74.3%, 26.7–63.4%, 39.7–69.8%, and 9.8–35.1%, respectively. Thus, there are no positive correlations between ACE-inhibitory activity and DH. A similar trend has been observed in the studies by Nielsen et al. [34] and Shu et al. [10] for milk protein-derived ACE-inhibitory peptides. In this study, the goat milk-derived hydrolysates was found to have a good ability to mediate an antihypertensive effect. Mullally et al. [35] found that the whey protein-derived hydrolysates had lower ACE inhibitory activity than the synthetic antihypertensive drug captopril. Unlike captopril, milk protein-derived ACE inhibitory peptides are expected to treat/prevent hypertension, since they have no undesirable side-effects. As a consequence, goat milk-derived ACE inhibitory peptides/hydrolysates may find an application as a nutraceutical in various physiologically functional foods.

## 4. Conclusions

In this study, skimmed cow milk and goat milk were enzymatic hydrolyzed by four different proteases including alkaline protease, trypsin, bromelain, and papain, and some properties of hydrolysates were analyzed. The DH values by four enzymes increased with hydrolysis time, and the highest value was obtained for alkaline protease-treated hydrolysates, which indicates that the alkaline protease has a more extensive ability to hydrolyze the milk proteins than other enzymes. Hydrolysates produced by alkaline protease exhibited the strongest ACE-inhibitory activity. The trends of ACE-inhibitory activity were almost unaffected by further increases in DH, which indicated that there was no relationship between the DH and ACE-inhibitory activity for milk proteins. Additionally, the ACE-inhibitory activity of hydrolysates from goat milk was higher than that of cow milk-derived hydrolysates. Therefore, goat milk is a good source of bioactive peptides with ACE-inhibitory activity, as compared to cow milk. Goat milk-derived ACE inhibitory peptides/hydrolysates may find applications as nutraceuticals in various physiologically functional foods. In order to obtain the functional dairy products with health-promoting effects, the generation and stability of the pertinent peptides during the proteolytic digestion of proteins in milk fermentation must be optimized.

## Figures and Tables

**Table 1:** Optimum conditions of enzymatic hydrolysis for various enzymes.

Enzyme	Optimum Conditions	
	Temperature °C	pH
Alkaline protease	45	9.5
Trypsin	37	8.0
Bromelain	45	6.0
Papain	37	7.0
